# Identification of serologic biomarkers for predicting microvascular invasion in hepatocellular carcinoma

**DOI:** 10.18632/oncotarget.7649

**Published:** 2016-02-23

**Authors:** Yuan-Quan Yu, Liang Wang, Yun Jin, Jia-Le Zhou, Yan-Hua Geng, Xing Jin, Xiao-Xiao Zhang, Jun-Jie Yang, Cheng-Ming Qian, Dong-Er Zhou, Da-Ren Liu, Shu-You Peng, Yan Luo, Lei Zheng, Jiang-Tao Li

**Affiliations:** ^1^ Department of Surgery, The Second Affiliated Hospital of Zhejiang University School of Medicine, Hangzhou, Zhejiang, China; ^2^ Department of Pathology, The Red Cross Hospital in Hangzhou, Hangzhou, Zhejiang, China; ^3^ Center for Cancer Research and Department of Biochemistry, Zhejiang University School of Basic Medical Sciences, Hangzhou, Zhejiang, China; ^4^ The Sidney Kimmel Comprehensive Cancer Center, The Johns Hopkins University School of Medicine, Baltimore, Maryland, USA

**Keywords:** hepatocellular carcinoma, microvascular invasion, sero-proteomics, biomarker, diagnosis

## Abstract

Microvascular invasion (MVI) of hepatocellular carcinoma (HCC) is a major risk factor for early recurrence and poor survival after curative surgical therapies. However, MVI can only be diagnosed by pathological examination following resection. The aim of this study is to identify serologic biomarkers for predicting MVI preoperatively to help facilitate treatment decisions. We used the sero-proteomic approach to identify antigens that induce corresponding antibody responses either specifically in the serum from MVI (+) patients or from MVI (−) patients. Six antigens were subsequently identified as HSP 70, HSP 90, alpha-enolase (Eno-1), Annexin A2, glutathione synthetase and beta-actin by mass spectrometry. The antibodies titers in sera corresponding to four of these six antigens were measured by ELISA and compared between 35 MVI (+) patients and 26 MVI (−) patients. The titers of anti-HSP 70 antibodies were significantly higher in MVI (−) patients than those in MVI (+) patients; and the titers of anti-Eno-1 antibodies were significantly lower in MVI (−) patients than those in MVI (+) patients. The results were subjected to multivariate analysis together with other clinicopathologic factors, suggesting that antibodies against HSP 70 and Eno-1 in sera are potential biomarkers for predicting MVI in HCC prior to surgical resection. These biomarkers should be further investigated as potential therapeutic targets.

## INTRODUCTION

Hepatocellular carcinoma (HCC) is the fifth most common malignancy and the third most common cause of cancer deaths worldwide [1]. The recurrence rate after curative surgeries remains as high as 70% following hepatectomy and 15%–30% following liver transplantation within 5 years, respectively [2]. Therefore, the treatment outcome of HCC remains unsatisfactory. A multitude of studies have suggested that the presence of microvascular invasion (MVI) in HCC is one of the most important risk factors for recurrence and metastasis in HCC following curative surgical resection or transplantation [3]. The latest AJCC staging system has adopted MVI as one of the parameters for the TNM staging of HCC [4].

It is reported that the rate of tumor recurrence within 1 year following curative resection in MVI (+) HCC patients was significantly higher than that in MVI (−) patients [3]. MVI is also a poor prognostic factor for recurrence and metastasis following liver transplantation for HCC [5]. The poor prognosis with MVI has raised the question on the benefit of the current surgical approach for MVI (+) HCC patients.

MVI was defined as the microscopic evidence of clusters of cancer cells observed in vessels located in the tumor capsule and/or in surrounding liver parenchyma [6]. MVI would not be detectable with the current radiologic imaging modalities [7]. MVI can only be detected by post-resection pathological examination. The application of MVI for guiding the selection of therapeutic strategies and predicting prognosis remains limited [8]. Recent studies suggested that tumor size, tumor number, degree of differentiation and serum level of des-gamma-carboxy prothrombin are the predictors for MVI [9, 10]. However, whether they are specific and independent predictive factors for MVI is questionable. Precancerous inflammation and immune response are known to play a critical role in the pathogenesis of HCC [11]. In addition, autoantibodies have been identified in the HCC patients [12]. Therefore, HCC associated tumor antigens may have induced antibody response in the sera. By identifying these antibody responses, we may identify biomarkers that are specifically associated with MVI. Thus, in this study, we used a sero-proteomic approach to identify HSP 70 and alpha-enolase (Eno-1) whose antibody titers in the sera are associated with the presence or the absence of MVI, respectively. This is the first time that serologic antibody markers have been identified as predictive biomarkers for MVI in HCC.

## RESULTS

### Clinicopathologic features

The clinicopathologic features of all 61 HCC patients with or without MVI are summarized in Table 1. Among these 61 patients, 35 patients' HCC had MVI and 26 patients' HCC did not have MVI. Serum hepatitis B antigen was positive in all of the patients. All the patients were Child-Pugh grade A at the time of surgery. The largest median tumor size was 4.5 cm (range 2.5–6 cm) in diameter. Multifocal tumors were present in 13 out of these 61 patients.

**Table 1:** Clinicopathologic features of the 61 HCC patients

Variables	Value
Age (year)	54.13 ± 12.31
**Gender**	
Males	54 (88.5%)
Females	7 (11.5%)
Largest tumor size (cm)	4.5 (2.5–6)
**Number of tumors**	
Single	48 (78.7%)
Multiple	13 (21.3%)
**Differentiation**	
Well	15 (24.6)
Moderate	34 (55.7)
Poor	12 (19.7)
**MVI**	
Negative	26 (42.6%)
Positive	35 (57.4%)

HCC, hepatocellular carcinoma; MVI, microvascular invasion.

### MVI (+) HCC tumor tissue is associated with unique antigens

**Figure 1:**
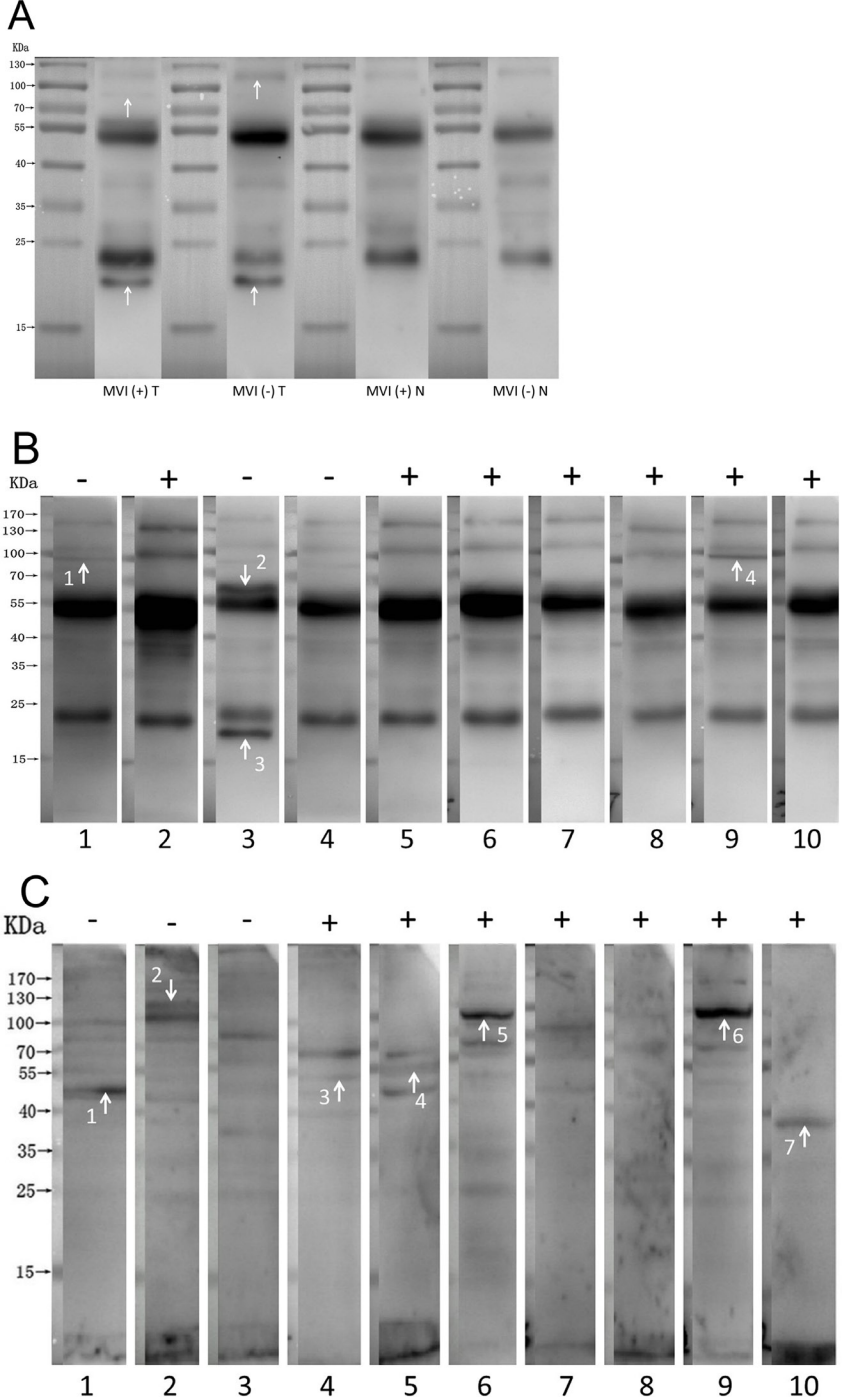
Antigen-antibody reactions between HCC lysates and serum (**A**) Autoantibodies are associated with both MVI (+) and MVI (−) HCC. Specific antigens are associated with HCC tumor tissue and MVI (+) HCC tumor tissue is also associated with unique antigens that are not present in MVI (−) HCC. Arrows indicate the specific antigens. MVI (+) T: lysates from tumor tissue of an MVI (+) HCC patient immunoblotted with serum from an MVI (+) HCC patient. MVI (−) T: lysates from tumor tissue of an MVI (−) HCC patient immunoblotted with serum from an MVI (+) HCC patient. MVI (+) N: lysates from paratumoral normal liver tissue of an MVI (+) HCC patient immunoblotted with serum from an MVI (+) HCC patient. MVI (−) N: lysates from paratumoral normal liver tissue of an MVI (−) HCC patient immunoblotted with the serum from an MVI (+) HCC patient. (**B**) Specific autoantibodies that recognize antigens on the HCC tissue are present in sera of MVI (+) or in those of MVI (−) patients. The lysate of tumor tissue from a MVI (+) HCC patient was immunoblotted with sera from MVI (+) HCC patients or MVI (−) HCC patients. Arrows indicate the distinguished protein bands comparing the blots between MVI (+) and MVI (−) groups. (**C**) Specific autoantibodies that recognize antigens on HCC cell line QGY-7703 are present in sera of MVI (+) or in those of MVI (−) patients. The lysate of QGY-7703 cells was immunoblotted with sera from MVI (+) HCC patients or MVI (−) HCC patients, respectively. Arrows indicate the bands of proteins that were specifically recognized by the serum from either MVI (+) or MVI (−) patients, but not from both.

We extracted cell lysates from tumor tissue and paratumoral normal liver tissue of an MVI (+) HCC patient and an MVI (−) HCC patient, respectively, and subjected it to the Western blot by the serum from an MVI (+) patient. Protein bands on the blot of MVI (+) tumor tissue were similar to, but not completely, the same as those on the blot of MVI (−) tumor tissue. As anticipated, protein bands on the blot of MVI (+) paratumoral normal liver tissue are also similar to those on the blot of MVI (−) paratumoral normal tissue. By contrast, bands are more obviously different between the blots of tumor tissue and paratumoral normal liver tissues (Figure 1A). These results suggested that specific antigens are associated with HCC tumor tissue and MVI (+) HCC tumor tissue is also associated with unique antigens that are not present in MVI (−) HCC.

### Specific autoantibodies appear to be present in sera of MVI (+) or in those of MVI (−) patients

We extracted cell lysates from tumor tissue of an MVI (+) HCC patient and subjected it to the Western blot by serum from 10 patients selected from the cohort randomly, including 7 MVI (+) and 3 MVI (−) patients, respectively. Specific autoantibodies appear to be present in sera of MVI (+) or in those of MVI (−) patients (Figure 1B).

We extracted cell lysates from HCC cell line QGY-7703 and subjected it to the Western blot by serum from the same 10 patients selected from the cohort randomly, including 7 MVI (+) and 3 MVI (−) patients, respectively. Using the proteome of HCC cell line as antigens, we also found that specific autoantibodies appear to be present in sera of MVI (+) or in those of MVI (−) patients (Figure 1C).

### Sero-proteomic identification of candidate MVI associated tumor antigens

**Figure 2:**
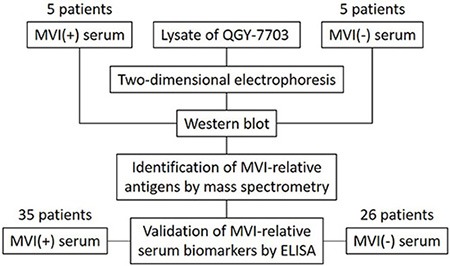
Schema of screening and validation of serum biomarkers for MVI The lysate of QGY-7703 is separated by two-dimensional electrophoresis followed by immunoblotting with the serum from 5 MVI (+) HCC patients or 5 MVI (−) HCC patients, respectively. The protein entities of spots that are either specifically associated with MVI (+) patients or with MVI (−) patients are identified by mass spectrometry. ELISA is performed to validate the presence of antibodies in the sera from 35 MVI (+) and 26 MVI (−) HCC patients that recognize MVI associated antigens.

**Figure 3:**
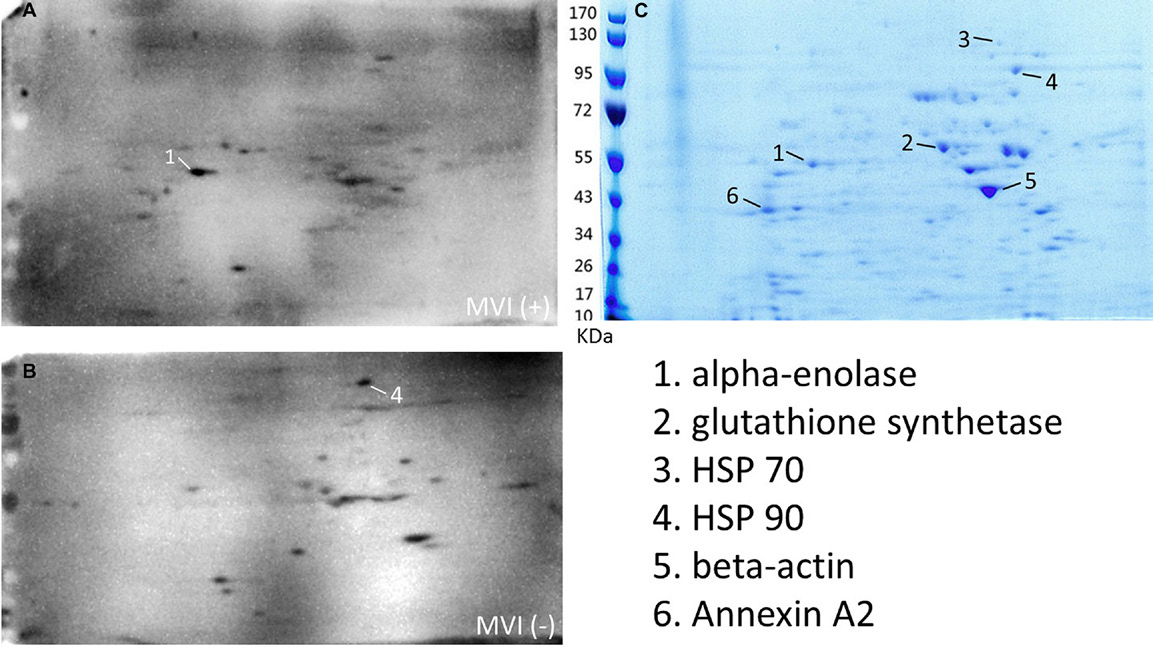
Sero-proteomic identification of antigens that induce autoantibodies specifically in either MVI (+) or MVI (−) patients (**A**) Multiple identical two-dimensional electrophoresis gels were run to separate the HCC cell lysate and transferred to the membrane. Membranes were blotted with the sera from 5 MVI (+) HCC patients, respectively. One representative blot was shown. (**B**) Membranes were blotted with the sera from 5 MVI (−) HCC patients, respectively. One representative blot was shown. (**C**) One two-dimensional electrophoresis gel was stained with Coomassie brilliant blue. Six spots were repeatedly seen in either the blot with MVI (+) sera or the blot with MVI (−) sera, but not both.

We thus attempted to identify the autoantibodies and their antigens associated with the status of MVI by following the sero-proteomic study schema described in Figure 2. The cell lysate from the QGY-7703 HCC cell line was subjected to two-dimensional electrophoresis (2DE). The 2DE gels were blotted by 5 MVI (+) sera and 5 MVI (−) sera, respectively. By comparing these blots, we identified that 2 spots were specifically recognized by MVI (−) sera and 4 spots were specifically recognized by MVI (+) sera, respectively. These spots were then analyzed by mass spectrometry (MS) for their protein identities. These six proteins are Eno-1, HSP 70, HSP 90, glutathione synthetase, beta-actin and Annexin A2 (Figure 3).

### Identification of serum anti-HSP 70 and anti-Eno-1 antibodies by ELISA as potential biomarkers for MVI

**Figure 4:**
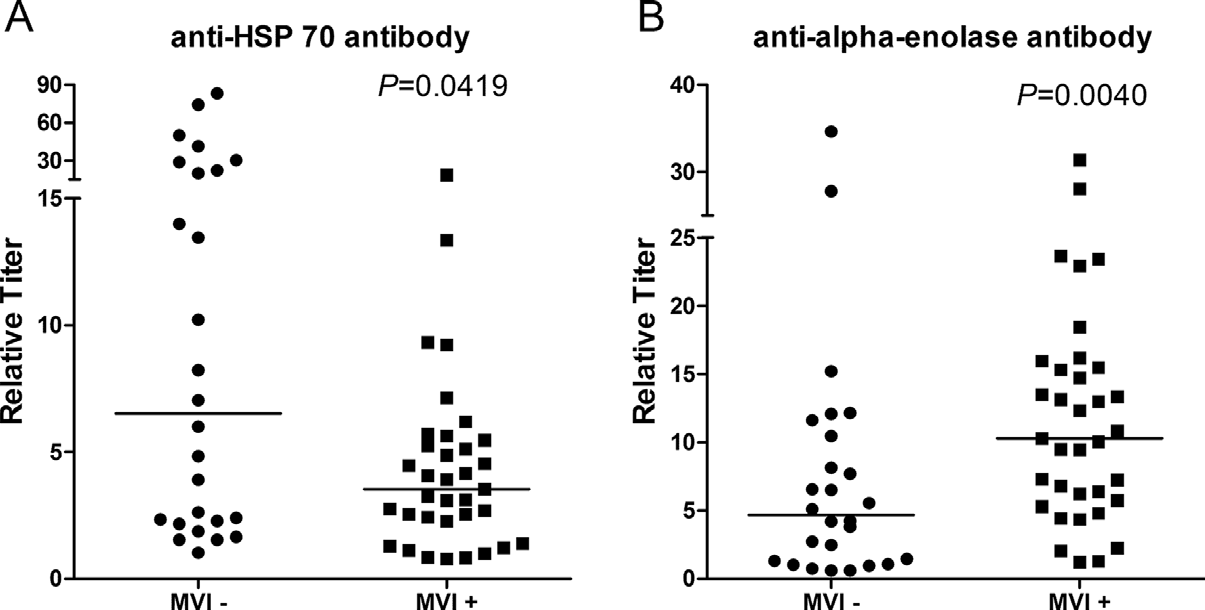
Quantification of the titers of anti-HSP 70 and anti-Eno-1 antibodies in the sera of MVI (−) and MVI (+) HCC patients by ELISA (**A**) Relative titers of anti-HSP 70 antibodies were significantly higher in MVI (−) HCC patients than those in MVI (+) HCC patients. (**B**) Relative titers of anti-Eno-1 antibodies were significantly lower in MVI (−) HCC patients than those in MVI (+) patients.

We then measured the relative titers of antibodies in patients' sera by using recombinant proteins in the ELISA assay. Among 6 proteins identified, 4 proteins have recombinant proteins available to us. We then measured the titers of antibodies against these four recombinant proteins by ELISA in sera from 26 MVI (−) and 35 MVI (+) HCC patients, respectively. The median relative titers of anti-HSP 70 antibody were 6.517 and 3.537 in the sera of MVI (−) and MVI (+) patients' serum, respectively. The relative titer of anti-HSP 70 antibody in the MVI (−) patients was significantly higher than that in the MVI (+) patients with a *p* value of 0.0419 (Figure 4A). The median relative titers of anti-Eno-1 antibody were 4.676 and 10.29 in sera of MVI (−) and MVI (+) patients' serum, respectively. The relative titer of anti-Eno-1 antibody in the sera of MVI (+) patients was significantly higher than that in the sera of MVI (−) patients with a *p* value of 0.0040 (Figure 4B).

We did not find a statistically significant difference of anti-HSP 90 antibody titer and anti-Annexin A2 antibody titer between MVI (+) and MVI (−) sera in the analysis of the initial 42 patients although a trend of difference toward anti-HSP 90 antibody titer was observed. We then measured the titers of anti-HSP 90 antibody in the sera of the entire cohort and found that anti-HSP 90 antibody titers are also significantly different between MVI (+) and MVI (−) sera (Supplementary Figure S1). Nevertheless, the association of MVI status and anti-HSP 90 antibody titer appears to be confounded by other clinicopathologic factors (Table 2). Therefore, we focus on HSP 70 and Eno-1 for further analysis.

**Table 2:** Multivariate analysis of clinicopathologic factors and predictive biomarkers potentially associated with MVI

Variables	OR	95% CI	*P* value
**Gender**			
male	Reference	Reference	0.501
female	3.391	0.097–118.608	
Age	1.073	0.961–1.197	0.209
**Number of tumor**			
single	Reference	Reference	0.999
multiple	1.002	0.107–9.359	
Tumor size	0.975	0.689–1.379	0.884
**Differentiation**			
well	Reference	Reference	–
moderate	0.320	0.005–20.047	0.589
poor	0.383	0.014–10.724	0.572
Titer of anti-HSP 70 antibodies	0.608	0.425–0.870	0.006
Titer of anti-HSP 90 antibodies	0.795	0.492–1.285	0.349
Titer of anti-Eno-1 antibodies	1.915	1.228–2.987	0.004

Eno-1, alpha-enolase; MVI, microvascular invasion.

We subsequently examined the expression of HSP 70 and Eno-1 in resected HCC specimens by immunohistochemistry and found that both HSP 70 and Eno-1 are expressed by HCC tumor cells, but minimally expressed by paratumoral normal hepatocytes (Supplementary Figure S2). This result suggests that the source of antigens that induce the anti-HSP 70 and anti-Eno-1 antibodies can be the HCC tumor cells.

### The predictive values of anti-HSP 70 and anti-Eno-1 antibodies titers for MVI

We then determined whether anti-HSP 70 and anti-Eno-1 antibodies titers in patients' sera can predict the MVI status. We performed both the univariate (Supplementary Table S1) and multivariate (Table 2) analyses. These analyses showed that the titers of anti-HSP 70 and anti-Eno-1 antibodies were independently associated with the status of MVI and thus are potential predictors for MVI.

**Figure 5:**
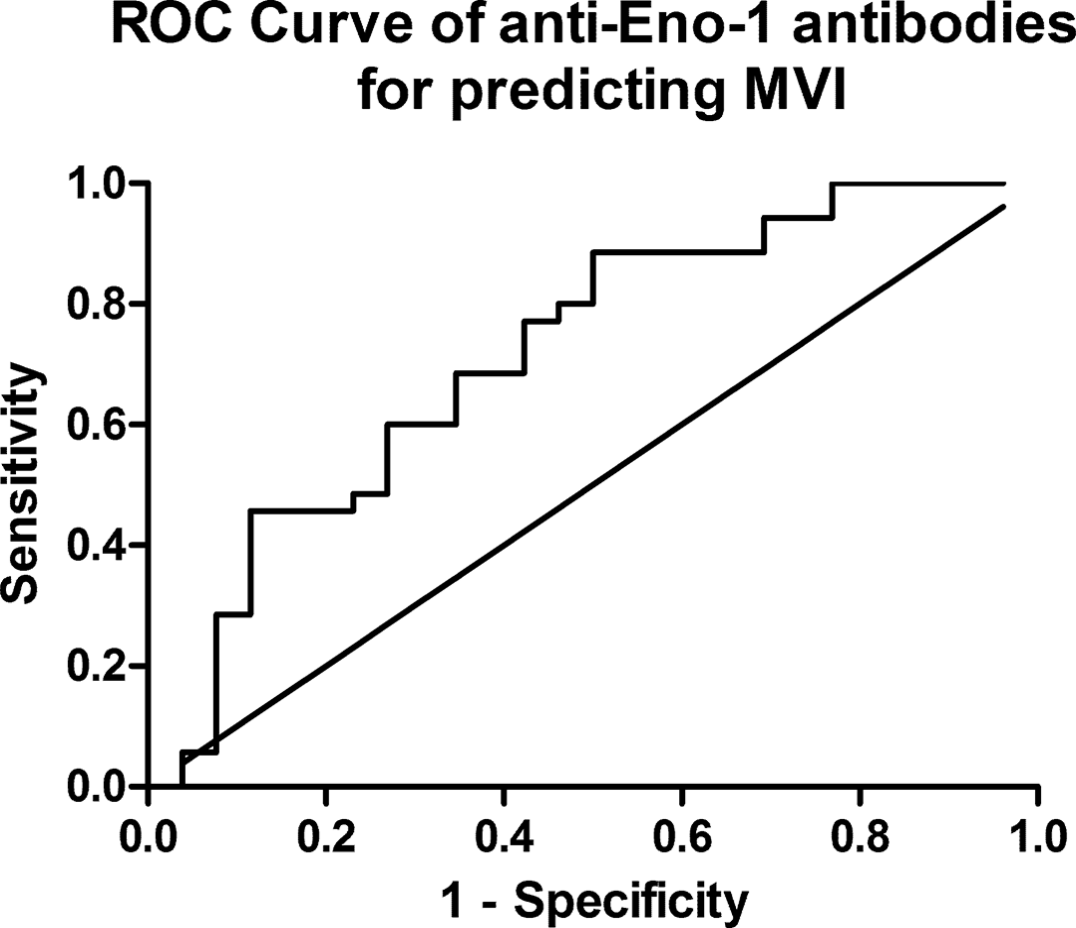
Receiver operating characteristic curve of relative titer of anti-Eno-1 antibodies The best cut-off value of titer of anti-Eno-1 is 4.301 with 88.57% sensitivity and 50% specificity, the area under the ROC curve is 0.7176.

The receiver operating characteristic (ROC) curve analysis showed that the cut-off value of titer of anti-Eno-1 antibody was 4.301 with 88.57% sensitivity and 50% specificity. The area under the ROC curve of Eno-1 was 0.7176 (Figure 5). The cut-off value of titer of anti-HSP 70 antibody for predicting MVI was 5.856 with 82.86% sensitivity and 53.85% specificity. The area under the ROC curve of anti-HSP 70 antibody titer was 0.6538 and smaller than that of anti-Eno-1 antibody (Supplementary Figure S3). Thus, the assay for the anti-HSP 70 antibody titer will need to be further improved.

## DISCUSSION

Our study has demonstrated that HCC tumor tissues express antigens that can be recognized by presumably autoantibodies in sera from HCC patients. Both MVI (+) tumor tissues and MVI (−) tumor tissues express unique antigens that are recognized by autoantibodies from HCC patients. Autoantibodies appear to be different in MVI (+) patients comparing to MVI (−) patients. These results suggest that one would potentially be able to identify specific autoantibodies that are associated with MVI (+) HCC or MVI (−) HCC.

Large vessel invasion can be recognized by the diagnostic imaging studies and often suggests that the patients would not benefit from upfront surgical treatments. These patients would often be treated by chemoembolization or radiation first. By contrast, MVI cannot be diagnosed preoperatively; however, it is known to be a poor prognostic factor for HCC following the current surgical treatments. Although these patients may still benefit from the surgery, neoadjuvant chemoembolization and/or radiation may be warranted to improve the outcome of the surgery. Therefore, a diagnostic biomarker to predict MVI preoperatively is highly demanded.

In this study, we identified autoantibodies against HSP 70 as a potential biomarker associated with MVI (−) HCC. Whether anti-HSP 70 antibodies can be used to predict MVI will need to be examined prospectively in a larger patient population. Members of HSP 70 family play a pivotal role in assisting protein folding, preventing protein aggregating and transporting proteins across membranes under physiological conditions. HCC overexpress HSP 70 in the cytosol, present it on their plasma membrane and can be actively secreted via lipid vesicles such as exosomes into the extracellular milieu [13]. It is reported that HSP 70 was able to elicit anti-tumor immunity [14]. The vesicular export of HSP 70 in extracellular fluids was reported to stimulate effector mechanisms of the immune system [15]. Thus, the presence of anti-HSP 70 antibodies in MVI (−) may be an antitumor protective mechanism that prevents the MVI (−) tumor from evolving into an MVI (+) one. Therefore, antitumor activities of anti-HSP 70 antibodies in the MVI (−) patients should be examined in the future. It is certainly possible that anti-HSP 70 antibodies do not have antitumor activities, but are a consequence of expression of HSP 70 by MVI (−) HCC. Then, HSP 70 itself should also be investigated as a potential biomarker of MVI (−) HCC.

In this study, we identified autoantibodies against Eno-1 as a potential biomarker associated with MVI (+) HCC. Whether anti-Eno-1 antibodies can be used to predict MVI will need to be examined prospectively in a larger patient population. Eno-1 is an isoenzyme of enolase, a key enzyme in the glycolytic pathway that catalyzes the conversion of 2-phosphoglycerate to phosphoenolpyruvate in the glycolytic pathway. Three enolase isoenzymes have been identified. The alpha form is present in most tissues and embryonically, the beta form is expressed in muscle tissues, and the gamma form, also known as neuron-specific enolase, is found only in neuronal tissues. Eno-1 has been detected not only in the cytoplasm but also at the membrane surface [16]. It was also shown that Eno-1 acts as the receptor of plasminogen [17, 18]. In tumor cells, the expression of Eno-1 was up-regulated and supported anaerobic proliferation (Warburg effect), driven tumor invasion through plasminogen activation and extracellular matrix degradation [17]. It was reported that down regulation of Eno-1 weakened invasiveness of the follicular thyroid carcinoma cell lines. Furthermore, exogenous Eno-1 expression promoted cancer cell proliferation, migration, invasion, and tumorigenesis. It has been reported that Eno-1 was over expressed in HCC cell lines [18]. Expression of Eno-1 was significantly higher in poorly differentiated HCC than in well-differentiated HCC. Expression of Eno-1 also correlated with tumor size and venous invasion [16]. Our study also shown that Eno-1 is a MVI associated antigens by showing the presence of anti-Eno-1 antibodies in MVI (+) HCC patients. Apparently, the current serum level of the anti-Eno-1 antibodies does not inhibit the occurrence of MVI. However, it will remain interesting to test if further enhanced production of anti-Eno-1 will inhibit MVI.

In summary, this study identified two autoantibodies as potential biomarkers for predicting MVI preoperatively. Further studies are warranted to establish the usage of the titers of anti-HSP 70 and anti-Eno-1 antibodies in sera to predict MVI. HSP 70 and Eno-1 are also potential markers of MVI. Anti-HSP 70 and anti-Eno-1 antibodies may have the potential of being developed into anti-HCC therapeutics.

## MATERIALS AND METHODS

### Patients and specimens

Between 2010 and 2015, HCC patients who underwent curative resection as the first line of therapy at The Second Affiliated Hospital of Zhejiang University School of Medicine were consented and enrolled in this study. Patients who received preoperative anti-cancer therapies had been excluded. Following the surgical resection, patients whose pathology examination presented macrovascular invasion were excluded. A total of 61 HCC patients were eligible. An experienced pathologist, who was blind to the patients' clinical histories, evaluated resected HCC specimens to identify MVI on H & E slides. All the patients were divided into MVI (+) and MVI (−) groups according to pathologic evaluation. This study was approved by the Ethics Committee of The Second Affiliated Hospital of Zhejiang University School of Medicine. Written informed consent was obtained from each patient. Clinicopathologic information of the 61 patients was collected. Serum samples were obtained from patients before surgery. HCC tissues and paraneoplastic liver tissues were obtained after resection immediately. Additional serums from 10 healthy individuals were collected at the time of routine physical examination, which were used in ELISA tests.

### Cell line

QGY-7703 cell line was purchased from the cell bank of the Chinese Academy of Sciences (Shanghai, China), which was maintained in RPMI-1640 media supplemented with 10% fetal bovine serum in a humidified incubator at 37°C and 5% CO_2_. The cell line QGY-7703 was developed from a 35-year-old Chinese female patient of primary hepatocellular carcinoma with a history of viral hepatitis [19].

### Western blot analysis

The total proteins of HCC tissues, normal liver tissues and QGY-7703 cells were extracted using a tissue protein extraction kit (CW Biotech, Beijing, China) supplemented with a complete protease inhibitor cocktail (Roche, Basel, Switzerland) respectively. The samples were homogenized and incubated on ice for 20 min followed by centrifugation at 10000 g at 4°C for 15 min. The supernatant was collected. Protein concentration was determined using a BCA protein assay kit (CW Biotech, Beijing, China). The protein sample was denatured in SDS-PAGE loading buffer in boiling water bath for 10 min. A total of 100 μg/lane proteins were separated by electrophoresis on a 10% sodium dodecylsulfate polyacrylamide gel. Then proteins were transferred onto PVDF membranes (Milipore, Billerica, MA, USA). The membranes were blocked in TBST containing defatted milk powder (5%, w/v) for 2 hours at room temperature (RT) and then incubated at 4°C with serum from MVI (−) or MVI (+) patient dilute in TBST containing bovine serum albumin (BSA) (5%, w/v) overnight. After rinsing in TBST, the membranes were incubated with HRP-conjugated goat-anti-human secondary antibody (1:5000 dilution; CW Biotech, Beijing, China) for 2 hours at RT. After rinsing again, membranes were detected by a chemiluminescence detection kit (Biological Industries, Kibbutz Beit Haemek, Israel). Signals were captured by ChemiDoc XRX system and analyzed by Quantity One software 4.6.2 (Bio-Rad, Hercules, CA, USA).

### Two-dimensional gel electrophoresis and immunoblotting

Sample preparation and 2DE was performed using ZOOM^®^ IPGRunner™ System (Invitrogen, Carlsbad, CA, USA) according to the manufacturer›s protocol. In brief, the QGY-7703 HCC cells were ultrasonically lysed in lysis buffer on ice. Supernatant was centrifuged at 16000 g for 30 min at 4°C. Protein concentration was measured by a protein assay kit. The concentration range is set as 8–10 mg/ml. The first-dimensional IEF was performed on a 7 cm pH 3–7 linear gradient strip. Each strip was rehydrated with 140 μl of the lysis buffer and 155 μl of the rehydration buffer overnight. IEF was run in four steps: 200 V for 20 min, 450 V for 15 min, 750 V for 15 min, and 2000 V for 30 min. The second-dimensional SDS-PAGE run was performed on a 4–12% Bis-Tris gel with 200 V for 40–50 min. After SDS-PAGE, one gel was stained with coomassie brilliant blue overnight. The proteins in another gel were transferred by electrophoresis to a PVDF membrane, and incubated with 150 μl sera from MVI (−) or MVI (+) patient diluted in 15 ml BSA solution (5%, w/v) overnight. Then, the PVDF membrane was incubated with HRP-conjugated goat-anti-human secondary antibody (1:5000 dilution; CW Biotech, Beijing, China) for 1 h at RT. The reaction was detected by a chemiluminescence detection kit (Biological Industries, Kibbutz Beit Haemek, Israel). Signals were captured by ChemiDoc XRX system and analyzed by Quantity One software 4.6.2 (Bio-Rad, Hercules, CA, USA).

### NanoLC-ESI-MS/MS analysis

The sample treatment had followed the commonly used protocol [20]. In brief, each protein sample was cleaned and digested in-gel with sequencing grade modified trypsin (Promega, Madison, WI, USA) in the digestion buffer (ammonium bicarbonate 100 mM, pH 8.5). The peptides from the digestion were extracted out with acetonitrile, and completely dried down in a SpeedVac device (Thermo, Waltham, MA, USA). The dried sample was then redissolved in sample solution (2% acetonitrile, 97.5% water, 0.5% formic acid). Each protein solution was reduced by dithiothreitol and all cysteine residues alkylated by iodoacetamide and cleaned. The sample was then digested with sequencing grade modified trypsin (Promega, Madison, WI, USA) in the digestion buffer (ammonium bicarbonate 100 mM, pH8.5). NanoLC-ESI -MS/MS analysis of digested protein samples was carried out by a high pressure liquid chromatography (HPLC) system (Agilent, Palo Alto, CA, USA) with a 75 micrometer inner diameter 8 cm in length reverse phase C18 column. The particle size of the C18 is 3 uM with pore size of 300 Ä. The injection time was 20 min. The HPLC Solvent A is 97.5% water, 2% acetonitrile, 0.5% formic acid. HPLC Solvent B was 9.5% water, 90% acetonitrile, 0.5% formic acid. The gradation time was 60 minutes from 2% Solvent B to 90% solvent B, plus 20 minutes for sample loading and 20 minutes for column washing. The column flow rate was around 800 nanoliter per minute after splitting. A typical injection volume was 3 μl. The HPLC system was on-line coupled with an ion trap mass spectrometer (LCQ DECA XP PLUS, Thermo, Waltham, MA, USA) in a way a sample eluted from HPLC column is directly ionized by a electrospray ionization process and enter into the mass spectrometer. The ionization voltage is often optimized each time and normally in a range of 1.2 kv–1.8 kv. The capillary temperature is set at 110°C. The mass spectrometer is set at the data-dependent mode to acquire MS/MS data via a low energy collision induced dissociation process. The default collision energy was 33% and the default charge state is 3. One full scan with 1 microscan with a mass range of 550 amu to 1800 amu was acquired, followed by one MS/MS scan of the most intense ion with a full mass range and three microscans. The dynamic exclusion feature is set as following: repeat count of 1 within 0.3 min and exclusion duration of 0.4 min. The exclusion width is 4 Da. As default, the mass spectrometric data was used to search against the most recent nonredundant protein database (NR database, NCBI) with ProtTech's ProtQuest software suite.

### Enzyme-linked immunosorbent assay (ELISA)

A previously published ELISA assay was used to detect antibodies in serum of HCC patients [21]. The calibration curve was generated with serum from 10 healthy individuals. The test was performed using 96-well plates. Wells were coated with 100 μl of recombinant Eno-1 [22] or HSP 70 (Sino Biological, Beijing, China) with a concentration of 1 μg/ml, or PBS alone for 1 h at RT. Peripheral wells were not used in order to avoid the edge effect. After three washes with PBST, the wells were blocked with 180 μl of 10% BSA at 4°C overnight. Then, the wells were incubated with 100 μl of serum diluted in PBS (1:800 and 1:1600) for 2 h at room temperature. After three washes with PBST, the wells were incubated with 100 μl of HRP-conjugated goat-anti-human secondary antibodies (1:30000 dilution; Sigma-Aldrich, St. Louis, MO, USA) for 1 h at RT. After three washes with PBST, the wells were then added with 100 μl of tetramethyl-benzidine solution (Sigma-Aldrich, St. Louis, MO, USA) for 20 min at RT away from light, and color development was stopped by addition of 50 μl of H_2_SO_4_ for 5 min. Absorbance was measured at 450 nm in a microplate reader. Optical density was calculated by subtracting the optical density measured in uncoated wells from that measured in coated wells.

### Statistical analysis

All statistical analyses were performed using statistical software package SPSS version 19.0 for windows (SPSS, Chicago, Illinois, USA). *P* values of < 0.05 were considered as statistically significant.

## SUPPLEMENTARY MATERIALS FIGURES AND TABLE



## Abbreviations

MVI: microvascular invasion
HCC: hepatocellular carcinoma
Eno-1: alpha-enolase
ROC: receiver operating characteristic
RT: room temperature
BSA: bovine serum albumin
2DE: two-dimensional electrophoresis
MS: mass spectrometry
HPLC: high pressure liquid chromatography
ELISA: enzyme-linked immunosorbent assay
