# Supplementary material for: Identification of serologic biomarkers for predicting microvascular invasion in hepatocellular carcinoma
Source: Oncotarget. 2016 Feb 23;7(13):16362–71. doi: 10.18632/oncotarget.7649 (PMC4941320; doi:10.18632/oncotarget.7649)
Supplement: Supplementary file 1 [file oncotarget-07-16362-s001.pdf]

Identification of serologic biomarkers for predicting microvascular invasion in hepatocellular carcinoma

Supplementary Materials

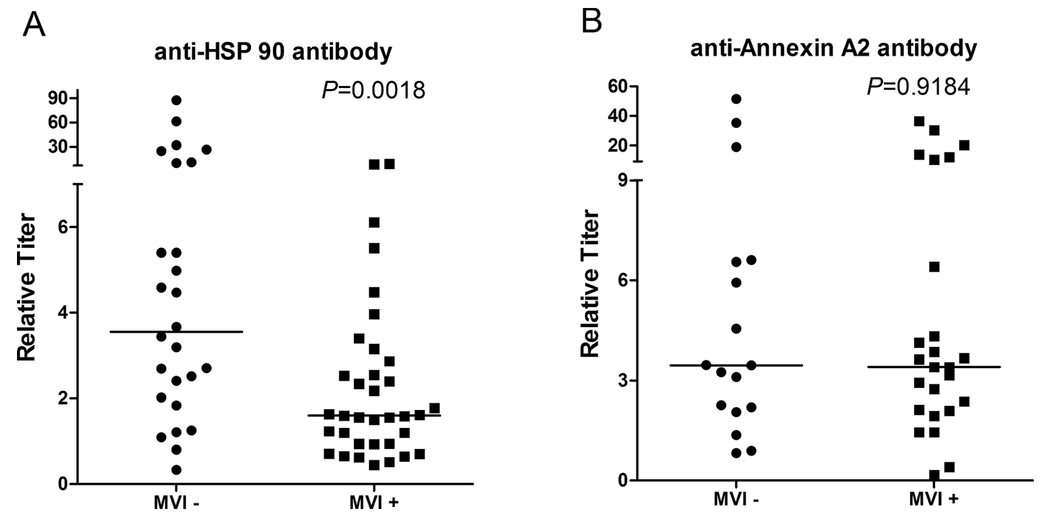

Supplementary Figure S1: Relative titers of anti-HSP 90 and anti-Annexin A2 antibodies in HCC patients' sera. (**A**) The median relative titers of anti-HSP 90 antibody were 3.555 and 1.596 in sera of MVI (−) and MVI (+) patients, respectively. The relative titer of anti-HSP 90 antibody in the MVI (−) patients was significantly higher than that in the MVI (+) patients with a *P* value of 0.0018. (**B**) The median relative titers of anti-Annexin A2 antibody were 3.451 and 3.401 in sera of MVI (−) and MVI (+) patients, respectively. There was no significant statistical difference in the titers of anti-Annexin A2 antibodies between MVI (+) and MVI (-) HCC patients.

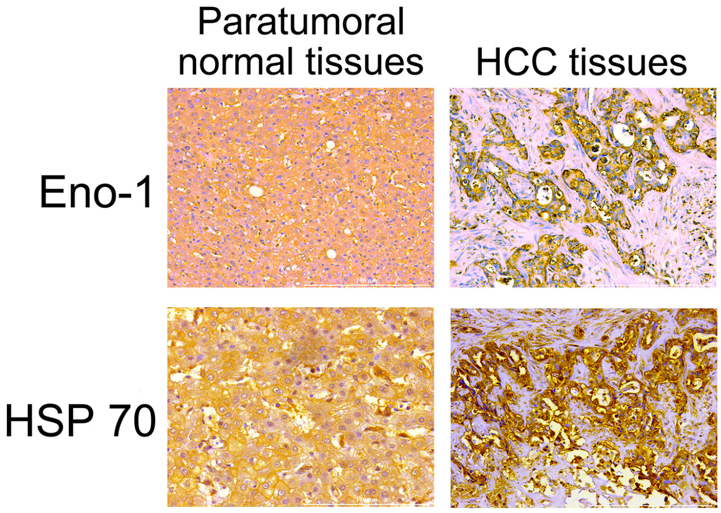

Supplementary Figure S2: Immunohistochemistry of Eno-1 and HSP 70 in HCC tissues comparing to paratumoral normal tissues. HSP 70 and Eno-1 are expressed in HCC tissues, but minimally in paratumoral normal tissues.

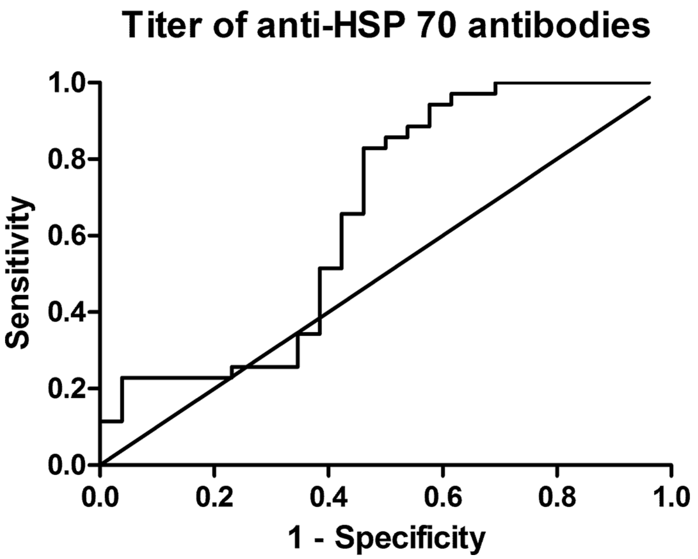

Supplementary Figure S3: The receiver operating characteristic curve of anti-HSP 70 antibodies for predicting MVI. The cut-off value of titer of anti-HSP 70 antibody for predicting MVI is 5.856 with 82.86% sensitivity and 53.85% specificity. The area under the ROC curve is 0.6538.

Supplemental Table S1: Univariate analysis of clinicopathologic factors and biomarkers potentially associated with MVI

Variables	MVI (−) (*n* = 26)	MVI (+) (*n* = 35)	*P* value
Gender			0.1251
Male	21	33	
Female	5	2	
Age (year)	54.85 ± 10.19	53.6 ± 13.8	0.6992
Number of tumors			0.7717
Single	20	28	
Multiple	6	7	
Largest tumor size (cm)	4.25 (2.5–6.125)	4.5 (2–6)	0.9767
Differentiation			0.3131
Well	6	9	
Moderate	17	17	
Poor	3	9	
Titer of anti-HSP 70 antibodies	6.517 (2.244–23.89)	3.537 (2.261–5.461)	0.0419
Titer of anti-HSP 90 antibodies	3.555 (1.975–10.200)	1.596 (0.94–2.866)	0.0018
Titer of anti-Eno-1 antibodies	4.676 (1.236–10.75)	10.29 (5.74–15.48)	0.0040
Titer of anti-Annexin A2 antibodies	3.451 (2.122–6.580)	3.401 (2.101–8.327)	0.9184

MVI, microvascular invasion. Eno-1, alpha-enolase.
